# Hearing aid fitting process in users fitted in a federal public institution: part II - self-assessment questionnaire results

**DOI:** 10.1016/S1808-8694(15)30127-0

**Published:** 2015-10-19

**Authors:** Carine Dias de Freitas, Maristela Julio Costa

**Affiliations:** a M.S in Human Communication Disorders Sciences - Federal University of Santa Maria - UFSM/RS, Speech and Hearing Therapist and Substitute Professor of Speech and Hearing Therapy of the Federal University of Santa Maria - UFSM/RS; b PhD in Human Communication Disorders Sciences/Speech and Hearing Therapy - Federal University of São Paulo - UNIFESP/SP. Speech and Hearing Therapy Adjunct professor of the Speech and Hearing Therapy - UFSM/RS

**Keywords:** hearing handicap, hearing disability, hearing aid, questionnaire

## Abstract

An efficient rehabilitation must be able to reduce impairment effects over the auditory and communication skills of individuals and promote psychosocial well being. **Aims:** check the feasibility of using self-assessment questionnaires and compare the results achieved by hearing aid fitting in users from a federal public institution, with and without complaints related to hearing amplification characteristics. **Materials and Methods:** 25 individuals, from 13 to 77 years of age, users of hearing aids. The HHIE-S/HHIA (Hearing Handicap Inventory for the Elderly Screening Version or for Adult) and APHAB (Abbreviated Profile of Hearing Aid Benefit) self-assessment questionnaires used with individuals without (Group 1) and with complaints related to amplification characteristics (Group 2). **Results:** we did not find significant differences between the HHIE-S/HHIA and APHAB groups; except in APHAB’s ease of communication item, where Group 1 seemed to benefit more. Moreover, we noticed a significant reduction in hearing disability with the use of hearing aids in favorable communication situations, noisy environments for both groups. **Conclusion:** these questionnaires proved to be valuable for predicting the difficulties faced by the users, and significant differences were found in favorable communication situations, where the group without complaints had the most benefit.

## INTRODUCTION

Hearing aid selection and fitting process, as well as their effective use, are fundamental in order to start hearing rehabilitation. In order to be considered efficient, it must be able to reduce the effects this disability brings over the auditory and communicative skills of the individual, in other words, his/her auditory disabilities and enhance their psychosocial well-being, thus reducing the disadvantages brought about by the disability or the auditory incapacity, called auditory handicap, a world wide used term by researchers, also used in this study. Moreover, these functional improvements must remain throughout time^1^.

In order to assess intervention results, the literature describes both objective procedures involving in situ measures, functional gains and formal speech recognition tasks, as subjective ones, which may concurrently show speech recognition and other aspects of life, such as difficulties in communication in daily activities. Thus, it is possible to check if the intervention process has reduced these disabilities or the auditory handicap, as well as check patient acceptance and satisfaction with the hearing aid^2^.

In order to investigate the individual’s performance or his perception of changes that may occur along time, favorable or not in hearing itself or in social or emotional relationships, the use of tools such as interviews and questionnaires are of the uttermost importance, because by using self-assessment questionnaires of disabilities or of auditory handicap, we obtain subjective measures based on the user’s judgment or his own perception^3^.

As referred to in the first part of this study, although many investigations have been carried out with hearing aid users, very few of them are attributed to users fit through the Public Health Care system. Therefore, the second part of this investigation aimed at:

1. Check the feasibility of using self-assessment questionnaires in patients from a public institution;
2. Compare hearing aid use results in users with and without complains related to the amplification characteristics.

## MATERIALS AND METHODS

This study was approved by the Ethics Committee of the Project Cabinet of the Health Sciences Center - CCS - UFSM, protocol # 112/2004. All participants signed the Informed Consent form in order to participate.

As it was described in the previous study, we interviewed 31 individuals fitted with hearing aids in the Hearing Aid Laboratory of the Federal University of Santa Maria, through the partnership signed between the State Health Secretariat of Rio Grande do Sul State and the Federal University of Santa Maria, in order to establish a joint action to provide hearing aid for the hearing handicap users of the Public Health Care System-SUS, under protocol 051/2000 signed in 12/29/2000 and published in the Federal Gazette on 02/08/2001, based on Ordinance 432 from the Ministry of Health.

After the interview, we started to assess the intervention results by means of subjective measures, that is, self-assessment questionnaires, which help us check the difficulties experienced in daily communication activities.

We excluded the cases in which the hearing aids were returned (2) and/or who had associated pathologies (4), which could interfere in evaluating the results.

The study group was made up of 25 users, 13 females and 12 males, with ages varying between 13 and 77 years, all of them with symmetric mixed bilateral or sensorineural hearing loss of moderate to severe levels, bilaterally fit with digital or computer-assisted programmable analogical hearing aids.

In order to assess communication difficulties and the social and emotional consequences of hearing disability in these users, we used the age-dependent Auditory Handicap self-assessment questionnaires for the elderly in the post-fitting period: Hearing Handicap Inventory for the Elderly Screening Version - HHIE-S (Annex 1), abridged version, developed in 19824 and adapted for the Portuguese Language in 1997^5^ and the Hearing Handicap Inventory for Adults - HHIA (Annex 2) for adults6, adapted for the Portuguese Language in 1998^2^.

Both the HHIE-S and the HHIA are questionnaires made up by two scales: one Social /Situational and the other one is emotional. The first aims at identifying the impact hearing loss would have on the activities performed by the individual, while the second one assesses the behavior and the emotional response to the hearing deficit.

HHIE-S is an abridged version of HHIE4, being faster and of easier understanding to be used in the elderly with hearing disability, made up of 10 questions broken down in five items for each scale. Now, the HHIA was developed from the HHIE to be used in hearing impaired patients with ages below 65 years, made up of 25 items, and 12 of them correspond to the Social/Institutional scale; and the other 13 are related to the emotional one. All the individuals below 65 years responded to the protocol adapted to the Brazilian Portuguese Language HHIA not excluding teenagers and young adults, because these are the only equivalent protocols to be used in different populations according to age range.

HHIA indices are identical to those from the HHIE-S. All the users were required to answer “yes” (4 points), “sometimes” (2 points) or “not” (no point) for each question. The answers obtained from patients with hearing aids were analyzed and the scores found by scale and total were standardized, in other words, turned into percentage indices, indicating its performance for this situation. The score value could vary between 0 and 100%, and the higher the score obtained; the higher was the patient’s self-perception of the handicap, 18 to 42% indicated little to moderate perception; and above 42%, indicated severe or significant perception.

ATTACHMENT I **HEARING HANDICAP INVENTORY FOR THE ELDERY SCREENING VERSION – HHIE-S**

**Abridged Version of the Auditory Handicap Questionnaire for the Elderly**

(Adapted from WIESELBERG, 1997)

INSTRUCTIONS: The following questionnaire has 10 questions. You should chose only one answer for each question, marking an (x) in the proper place. Some questions are similar to others, but they actually have subtle differences, which allow us to better assess the answers. There is no right or wrong answer. You should mark the one you believe to be more adequate in your case or situation.

Thank you for your participation!

**Table:** 

	Yes	Sometimes	No
E-1. Does your hearing difficulty make you embarrassed or out of place when you are introduced to strangers?			
E-2. Does your hearing difficulty make you feel frustrated or unhappy when you talk o others in your family?			
S-3. Do you feel it is harder to hear when the other person is whispering?			
E-4. Do you feel cheated because of your hearing problem?			
S-5. Does your hard of hearing make things difficult when you visit relatives or neighbors?			
S-6. Does your hearing impairment prevent you from going more often to religious services?			
E-7. Does your hearing impairment make you have arguments or fights with your family?			
S-8. Does your hearing difficulty make it difficult for you to watch TV or listen to the radio?			
E-9. Do you think your hearing difficulty somehow limits your personal or social life?			
S-10. Does your hearing impairment bring you any difficulty when you are in a restaurant with family or friends?

In a second phase, in order to assess hearing aid benefit, we used the Abbreviated Profile of Hearing aid Benefit - APHAB7 (Annex 3) adapted to the Portuguese Language in 1997^8^.

APHAB is a self-assessment questionnaire, useful to quantify the disability associated with the hearing loss and its reduction with the use of sound amplification. It is based on 24 items, distributed in four subscales, namely: Ease of communication (EC), Reverberation (RV) and Environmental Noise (EN), made to assess speech understanding in different situations of our daily lives, and also Sound Aversion (SA), which quantifies the negative reactions to environmental sounds. For each item we offered two answer options, one “without hearing aids” and another “with hearing aids”, through which we could assess both the individual’s own performance with and without the hearing aid with the benefit supplied by amplification, factoring in the difference between these two indices.

Users were instructed to answer the same item in each subscale, for both options “with” and “without” hearing aids, selecting the answer within a continuous seven points scale (A, B, C, D, E, F, G), which should indicate how frequent each statement was true. Each answer option was associated with a descriptive term and to a percentage, which are: A “always” (99%), B “almost always” (87%), C “usually” (75%), D “half of the time” (50%), and “sometimes” (25%), F “rarely” (12%) and G “never” (1%).

The response from each individual for each of the subscales was analyzed and calculated by means of a computer program “Phonak Fitting Guideline 8.5”, indicating its performance for each situation “without” and “with” hearing loss and its benefit, calculating the differences between responses for each situation.

In order to analyze the results attained, considering each subscale individually, it is necessary that we have a minimum difference of 22% between the indices with and without hearing aid in at least one of the subscales to represent a real difference between the two situations. Now, if the goal is to have a global evaluation of amplification, a hearing aid index of 10% better than that without hearing aid in three subscales: FC, RV, and RA, really represents an improvement in the individual’s performance^9^.

The first questions in the questionnaire were asked by the examiner and the following, whenever possible, were answered by the individual being studied. In those users in whom we noticed some type of difficulty, either understanding or expressing written language, the examiner asked all the questions verbally.

We separated the individuals assessed in two groups based on the qualitative results related with the amplification previously investigated, as follows:

Group 1 (G1) Without complaints related to the amplification characteristics (N=8).
Group 2 (G2) With complaints related with amplification characteristics (N=15).

ATTACHMENT II **HEARING HANDICAP INVENTORY FOR ADULTS – HHIA**

**Questionnaire for the Assessment of Auditory Handicap in Adults**

(Adapted from ALMEIDA, 1998)

INSTRUCTIONS: The following questionnaire has 25 questions. You should pick only one answer for each question, by placing an (x) in the one you find more adequate. Some questions are similar to others, but they actually bear subtle differences that allow us to better evaluate the answers. There is no right or wrong answer. You should mark the one you think is the most adequate to your case or situation.

Thank you for participating!

**Table:** 

	Yes	Sometimes	No
S-1. Does your hearing difficulty make you use the telephone less than you wish you could?			
E-2. Does your hearing difficulty make you feel embarrassed or out of place when you are introduced to strangers?			
S-3. Does your hearing difficulty make you avoid groups of people?			
E-4. Does your hearing difficulty make you touchy?			
E-5. Does your hearing difficulty make you feel frustrated or unhappy when you talk to your family?			
S-6. Does your hearing impairment makes things difficult for you when you go to a party or a social gathering?			
E-7. Does your hearing difficulty make you feel frustrated when you talk to work mates?			
S-8. Do you feel hard of hearing when you go to the movies or to the theater?			
E-9. Do you feel harmed or a lesser person because of your hearing impairment?			
S-10. Does your hearing impairment cause you difficulties when you visit friends, relatives or neighbors?			
S-11. Does your hearing difficulty cause you problems to hear/understand your work mates?			
E-12. Does your hearing difficulty make you nervous?			
S-13. Does your hearing difficulty make you visit friends, relatives or neighbors less often than you wish you could?			
E-14. Does your hearing difficulty make you fight or argue with your family?			
S-15. Does your hearing impairment make it hard for you to watch TV or listen to the radio?			
S-16. Does your hearing impairment make you go shopping less often than you wish you did?			
E-17. Does your hearing difficulty make you somehow sad or bored?			
E-18. Does your hearing difficulty make you lonesome?			
S-19. Does your hearing difficulty make you want to talk less with the people in your family?			
E-20. Do you think your hearing difficulty somehow impairs or limits your personal or social life?			
S-21. Does your hearing impairment cause you difficulty when you are in a restaurant with friends or family?			
E-22. Does your hearing difficulty make you feel sad or depressed?			
S-23. Does your hearing difficulty make you watch TV or listen to the radio less then you wish you could?			
E-24. Does your hearing difficulties make you feel embarrassed or less comfortable when you talk to friends?			
E-25. Does your hearing difficulty make your feel isolated or “left aside” when in a group of people?

ATTACHMENT III **ABBREVIATED PROFILE OF HEARING AID BENEFIT – APHAB**

**Assessment Protocol of the Hearing Aid Benefit**

(Adapted by ALMEIDA, GORDO, IÓRIO and SCHARLACH, 1997)

INSTRUCTIONS: Please, circle the answers that get closer to your day-to-day. Notice that each choice you make includes a percentage. You can use this to decide upon your answers. For example, if one item is true for about 75% of the times, circle letter C. If you have not experienced the situation described, try to thing of a similar situation. If you have no idea, leave it without answering. If you have not experienced the situation described, try to think of a similar situation. If you are still clueless, leave it without answering. A Always (99%) B Almost always (87%) C Usually (75%) D Half of he times (50%) E Sometimes (25%) F Rarely (12%) G Never (1%).

**Table:** 

		Without hearing aid	With hearing aid
1.	When I am in the supermarket, talking to the cashier, I can follow the conversation.	A B C D E F G	A B C D E F G
2.	I miss information when I attend classes, courses or talks.	A B C D E F G	A B C D E F G
3.	Unexpected sounds such as car alarms are uncomfortable.	A B C D E F G	A B C D E F G
4.	I have difficulties in hearing the conversation with my family at home.	A B C D E F G	A B C D E F G
5.	I have difficulties to understand a dialogue in the cinema or in the theater.	A B C D E F G	A B C D E F G
6.	When I am listening to the news in the car radio and other family members are talking, I have difficulty to understand what is being said.	A B C D E F G	A B C D E F G
7.	When I am in a dinner table with many people and I am trying to talk with one of them, it is difficult to understand their talk.	A B C D E F G	A B C D E F G
8.	Sounds from traffic are very intense.	A B C D E F G	A B C D E F G
9.	When I am talking o someone in a large empty room, I understand the words.	A B C D E F G	A B C D E F G
10.	When I am in a small room, asking or answering questions, I have difficulties to follow on the conversation.	A B C D E F G	A B C D E F G
11.	When I am in a theater or at the cinema watching a play or a movie, people around me are whispering or crunching.	A B C D E F G	A B C D E F G
12.	When I am talking in a low voice with a friend, I have difficulties to understand.	A B C D E F G	A B C D E F G
13.	The sounds of running water, such as from the kitchen tap, in the bathroom or in the shower are uncomfortable or intense.	A B C D E F G	A B C D E F G
14.	When a speaker addresses a small group of people and everyone is listening attentively, I have to strain myself in order to understand.	A B C D E F G	A B C D E F G
15.	When I am talking with my physician in the examination room, it is difficult to follow on the conversation.	A B C D E F G	A B C D E F G
16.	I can understand the conversation even when many people are talking at the same time.	A B C D E F G	A B C D E F G
17.	Construction work noise is uncomfortably loud.	A B C D E F G	A B C D E F G
18.	It is difficult for me to understand what is said in talks or in church.	A B C D E F G	A B C D E F G
19.	I can communicate with others when I am in a crowd.	A B C D E F G	A B C D E F G
20.	The sound of a nearby siren is so loud that I need to cover my ears.	A B C D E F G	A B C D E F G
21.	I can follow the words of a sermon in mess or in a religious cult.	A B C D E F G	A B C D E F G
22.	The sound of a car breaking is uncomfortably loud.	A B C D E F G	A B C D E F G
23.	Talking to another person in a silent room, I need to ask him/her to repeat.	A B C D E F G	A B C D E F G
24.	I have difficulties to understand what the others are saying when the air conditioning or the fan is on.	A B C D E F G	A B C D E F G

Afterwards, the results were statistically analyzed, by using a non-parametric test, which was the Krukal-Wallis test to analyze the statistically significant differences between the situations with and without hearing aids in the APHAB questionnaire and between the two groups by means of HHIE-S, HHIA and APHAB. The level of rejection towards the null hypothesis was fixed in a value equal to or less than 5%. Statistically significant results were marked with an asterisk (*).

## RESULTS

1. Handicap Inventory for the Elderly Screening Version - HHIE-S or Hearing Handicap Inventory for Adult - HHIA Results obtained for Groups 1 and 2.

On Table 1 one can see the averages, the standard deviation and the minimum and maximum percentage values per scale (Social/Situational and Emotional) and total, obtained by means of these HHIE-S or HHIA auditory handicap results (%) in a post fitting period, in Groups 1 and 2 like the results of the statistical analysis.

**Table 1:** Comparative analysis of the mean percentage values obtained from the HHIE-S or HHIA questionnaires (%) in Groups 1 (N=8) and 2 (N=17).

	HHIE-S/HHIA (%)					
	Social/Situational		Emotional		Total	
	G 1	G 2	G 1	G 2	G 1	G 2
Mean	17,50	24,18	17,25	21,65	34,75	45,82
Standard Deviation	9,58	11,66	9,82	12,66	17,47	20,96
Minimum	4,00	8,00	0,00	0,00	4,00	20,00
Maximum	28,00	50,00	29,00	45,00	54,00	95,00
p- value	0,2671		0,4311		0,3359	

There is no statistically significant difference - Krukal-Wallis test (p > 0.05).

**Figure 1:**
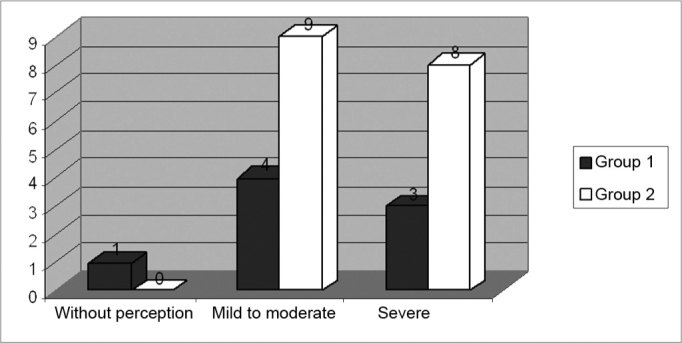
Distribution by degrees of perception of the auditory handicap for Groups 1 (N=8) and 2 (N=17).

Figure 1 shows the three levels of the auditory handicap perception, according to distribution for the Groups 1 (N = 8) and 2 (N = 17).

2. Abbreviated Profile of Hearing Aid Benefit - APHAB results obtained for Groups 1 and 2.

Table 2 shows the averages, standard deviations and minimum and maximum percentage values of auditory difficulties for each subscale: Ease of Communication (EC), Reverberation (RV), Environmental Noise (EN) and Sound Aversion (SA), obtained after hearing aid fitting from the APHAB questionnaire, in those from Group 1 (N = 8), for situations with and without hearing aids, as well as the result from the statistical analysis.

**Table 2:** Comparative analysis of the percentages obtained from the APHAB questionnaire (%) without and with hearing aids in Group 1 (N=8).

				APHAB (%)				
	FC		RV		RA		AS	
	Without	With	Without	With	Without	With	Without	With
Mean	77,5	8,87	63,75	26,63	71,63	23,25	21,25	30,5
Standard Deviation	18,49	8,41	16,77	11,16	22,24	13,96	34,33	34,05
Minimum	49,00	1,00	35,00	17,00	46,00	1,00	1,00	1,00
Maximum	99,00	21,00	82,00	48,00	99,00	39,00	93,00	90,00
p- value	0,0008*	0,0019*	0,0008*	0,2410				

* There is statistically significant difference – Kruskal-Wallis test (p > 0.05).

Table 3 shows the averages, standard deviations and minimum and maximum percentage values of the hearing difficulties for each subscale: Ease of Communication (EC), Reverberation (RV), Environmental Noise (EN) and Sound Aversion (SA), obtained after hearing aid fitting from the APHAB questionnaire in those patients from Group 2, for situations with and without hearing aids (N = 17), as well as the result from the statistical analysis.

**Table 3:** Comparative analysis of the percentages obtained from the APHAB questionnaire (%) without and with hearing aids in Group 2 (N=17).

				APHAB (%)				
	FC		RV		RA		AS	
	Without	With	Without	With	Without	With	Without	With
Mean	68,65	29,06	71,06	32,94	67,18	27,29	10,94	39,18
Standard Deviation	24,34	29,06	18,65	32,94	14,67	27,29	16,69	39,12
Minimum	8,00	1,00	29,00	17,00	33,00	1,00	1,00	5,00
Maximum	99,00	76,00	99,00	66,00	84,00	52,00	54,00	95,00
p- value	0,0001*		0,0001*		0,0001*		0,0008*	

* There is statistically significant difference – Kruskal-Wallis test (p > 0.05)..

Table 4 shows the averages, standard deviation and maximum and minimum percentage values regarding the benefit, coming from the difference in results between the situations with and without the hearing aid for each subscale: Ease of Communication (EC), Reverberation (RV), Environmental Noise (EN) and Sound Aversion (SA), obtained after fitting by means of the APHAB questionnaire (%) in Groups 1 and 2, as well as the result from the statistical analysis.

**Table 4:** Comparative analysis of the percentages obtained from the APHAB questionnaire (%) between Groups 1 (N=8) and 2 (N=17).

				APHAB (%)				
	FC		RV		RA		AS	
	G 1	G 2	G 1	G 2	G 1	G 2	G 1	G 2
Mean	68,62	39,59	37,12	38,12	48,38	39,88	-9,25	-28,18
Standard Deviation	21,73	35,99	23,23	23,81	31,73	23,08	44,39	27,62
Minimum	37,00	-68,00	-13,00	-8,00	9,00	-17,00	-73,00	-79,00
Maximum	98,00	90,00	65,00	74,00	83,00	71,00	85,00	31,00
p- value	0,0230*	0,9534	0,4487	0,2671				

* There is statistically significant difference – Kruskal-Wallis test (p > 0.05).

**Figure 2:**
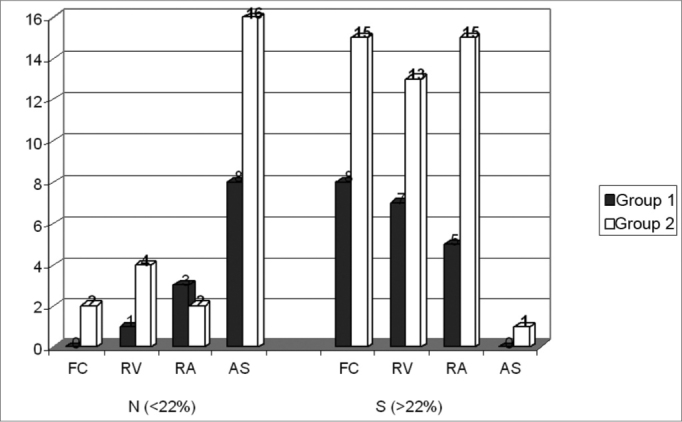
Distribution of the benefit by subscale for Groups 1 (N=8) and 2 (N=17).

Figure 2 shows the benefit distribution by subscales for Groups 1 (N = 8) and 2 (N = 17).

**Figure 3:**
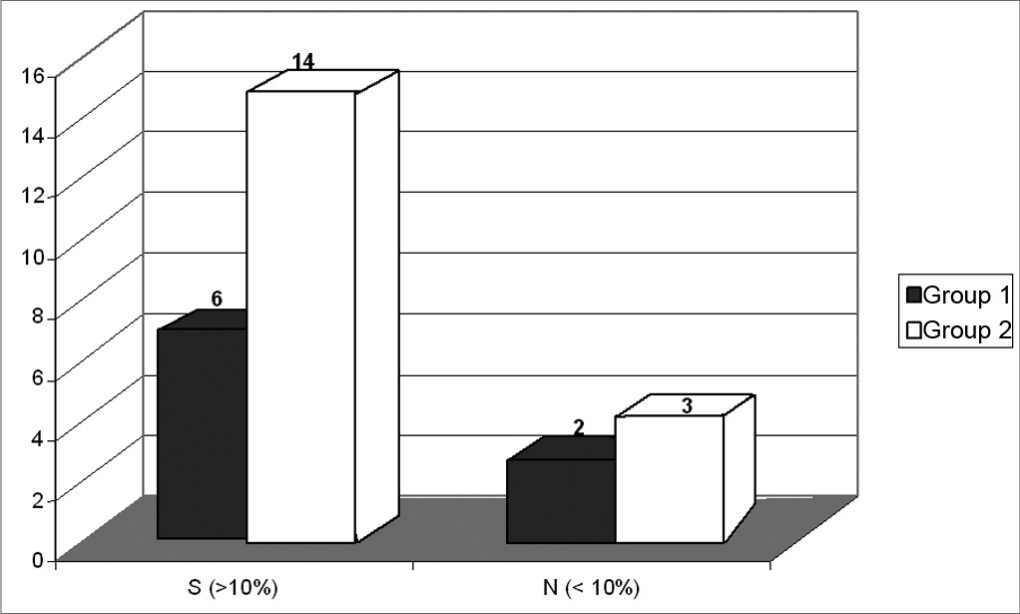
Distribution of the global benefit for Groups 1 (N = 8) and 2 (N = 17).

Figure 3 shows the distribution of global benefit, significant (S) or not (N) for Groups 1 (N = 8) and 2 (N = 17).

## DISCUSSION

1. Comments on the results from the Handicap Inventory for the Elderly Screening Version - HHIE-S or Hearing Handicap Inventory for Adult - HHIA obtained for Groups 1 and 2.

We noticed a 34.75% of average percentage total scale index for Group 1, with a variation range between 4% and 54%. In the social/situational scale we found an average index of 17.50% and in the emotional scale it was of 17.25%. For Group 2, there was an average index for the total scale of 45.82%, varying between 20% and 95%, and from the total, 24.18% corresponded to the social /situational scale and 21.65%, to the emotional (Table 1).

Statistically significant differences for the social/situational scale, emotional and total between the groups were not found when compared to the indices of auditory handicap perception. Nonetheless, we see better results in the social/situational, emotional and total scales for those patients in Group 1 when compared to Group 2, in other words, there is less auditory handicap perception in the group which did not present complaints related to amplification characteristics (Table 1). With this, we notice the importance of utilizing these questionnaires, which make it possible to investigate the patient’s perception about his/her own communication difficulties, aiding in monitoring in time and identifying their real auditory needs besides those which are possible to be seen in routine audiologic evaluations^10^, ^11^.

Of the 8 users who made up Group 1 in this study, 1 (12.5%) did not show any self-perception of an auditory handicap with scores below 16%; 4 (50%) had self-perception of mild to moderate handicap corresponding to an interval of 18 to 42%; and 3 (37.50%) showed a significant self-perception of the handicap imposed by the auditory deficiency and/or disability, with indices above 42% (Figure 1). Meanwhile, of the 17 individuals from Group 2, not one of them had any perception of their auditory handicap^3^. However, 9 (52.94%) had self-perception from mild to moderate and 8 (47.02%) presented with significant auditory handicap (Figure 1).

Many investigations were carried out in the post fitting period, with the goal of checking the benefit in the long run, of which results from the handicap self-perception found throughout the three years of sound amplification use are similar to those found in the present study, in other words, a mild to moderate perception of the auditory handicap.^12^, ^13^, ^14^

According to the aforementioned studies, we notice that the benefit and the satisfaction obtained from sound amplification after a short time interval can show a marked reduction in auditory handicap thanks to the excitement and high user’s expectation, however, a real improvement in performance, able to assess amplification limitations must be obtained after at least 3 months of use, which should remain and stabilize with time.

Thus, patient follow up could in fact show treatment efficacy and also check if the amplification would continue being considered beneficial or not1. Thus, these tools, among their many utilities, can be considered useful to check communication problems and/or the psychosocial consequences of hearing loss which remain, even with sound amplification, helping patients through the fitting process, which does not end after checking results, it lasts through time.

2. Comments on the results of the Abbreviated Profile of Hearing Aid Benefit - APHAB obtained for Groups 1 and 2.

On Tables 2 and 3 average percentage indices obtained through the application of the APHAB questionnaire for each subscale, without a hearing aid, which were for Group 1: 77.5% (FC), 63.75% (RA), 71.63% (RV) and 21.25% (AS), and for Group 2: 68.65% (FC), 71.06% (RA), 67.18% (RV) and 10.94% (AS). And for the situation with hearing aids, in Group 1, the average percentage indices observed were less in the subscales, namely: 8.87% (FC), 26.63% (RA), and 23.25% (RV). The same thing did not occur in subscale AS, in which we obtained the worst result with hearing aids equal to 30.5% (Table 2). As far as Group 2 is concerned, average indices with hearing aid were also lower for subscales FC, RA and RV, presenting indices equal to 9.06%, 32.94% and 27.29%, respectively. Subscale AS presented higher values with hearing aid, with average percentage index equal to 30.18% (Table 3).

Therefore, statistically significant differences among he results obtained with and without hearing aids for subscales FC, RV and RA were found for both groups (Table 2 and 3), and performance was better with hearing aids. However, for subscale AS, which encompasses the negative aspects related to environmental sound perception, we did not see statistically significant difference for Group 1 between the two situations (Table 2), while Group 2 revealed a statistically significant difference between the two situations (Table 3), however, performance was worse with hearing aids.1

Such results can be expected, having seen that hearing aids aid in verbal communications under favorable conditions and even in not very pleasant communication situations; nonetheless, with high levels or just a mild worsening in subscale AS with hearing aids can be justified by the fact that the acoustic signals become more intense with sound amplification, thus causing negative reactions to environmental sound. Similar findings to these ones were encountered in many studies^7^, ^2^, ^15^.

The benefit reflected by the use of sound amplification was calculated by the differences between the APHAB questionnaire response for both situations, namely: with and without the hearing aid. Positive benefit values mean that a better performance was seen with hearing aid when compared to those without hearing aid. Conversely, negative values show a perception of a worse performance with hearing aid when compared to without hearing aid.

For Group 1, we found benefit values of 68.62% (FC), 48.38% (RA), 37.12% (RV) and -9.25% (AS) and in Group 2, we noticed indices of 39.59% (FC), 39.88% (RA), 38.12% (RV) and -28.18% (AS), thus showing a significant reduction in hearing loss with the use of hearing aids in favorable communication situations (FC), in reverberating environments (RV) and in the presence of environmental noise (RA) for both groups. As previously stated, in negative perception situations, performance with the hearing aid was worse for both groups, confirmed by negative benefit indices in Subscale AS (Table 4).

By analyzing the results we noticed statistically significant differences between Groups 1 and 2 only in subscale FC, and Group 1 showed the best results. Nonetheless, in non-favorable verbal communications, we had better benefit results in subscale RV for Group 1 when compared with Group 2, and similar results between both groups in subscale RA (Table 4).

In subscale AS we did not find statistically significant differences between average indexes from Groups 1 and 2, nonetheless, it is stressed that Group 1 was the one who had the lowest results, suggesting that the complaints related to the amplification characteristics presented by Group 2 influenced the performance with hearing aids in high intensity environmental sound, because many of the complaints were related to the high intensity of environmental sounds - headaches and discomfort towards sounds.

Different research projects^16^, ^17^, ^18^, ^19^, ^20^, ^21^, ^22^, ^23^ were conducted in an attempt to investigate and compare the benefit attained by means of applying self assessment questionnaires with different hearing aid technologies bearing different electro acoustic characteristics, though none of them had reported hearing problems after hearing aid fitting with the results found in this hearing impairment self-assessment protocol.

Despite all of this, as we compared benefit results by scale in this study with those from other studies which assessed experienced users, we found for both groups 1 and 2 similar results as far as benefit is concerned equal to 35%7and 41.42%^2^. In subscale RA, the results we found are higher when compared to those found in the first study mentioned, which was 36.74%, Group 1 showed higher results, and Group 2 showed similar results. In favorable communication situations, only Group 2 presented benefits similar to 42.89%^2^, which is higher when compared to 31%7. Group 1 showed higher benefit rates when compared to the ones aforementioned. We believe that this is related to the fact that Group 1 was well adapted and without complaints related to amplification characteristics.

We found negative results in the aforementioned studies as far as the sound aversion scale is concerned, representing the worst results found in this study when compared to those from Group 1, and when compared to Group 2 they are similar to -30%7 and greater than -18.11%^2^.

We recommend that the indices of subscale AS be the lowest possible, that is, closer to zero, indicating that the sound amplified by the hearing aids should not be uncomfortably loud, because we believe that this subscale can provide information about adapting the maximum output of these hearing aids, although new investigations are necessary to use it in a proper and precise way.^9^ With this, we can explain the fact that Group 1 presented indices closer to zero when compared to Group 2, since these patients do not complain about the amplification characteristics, which is different from Group 2, very likely because the complaints reported presented higher negative values, which are far from the ideal.

As we perform an individual analysis of the benefit’s results, we observed a difference higher than 22%, showing a significant benefit per subscale in 100% (8), 87.5% (7) and 62.5% (5) of users from Group 1 in the respective subscales FC, RV and RA. On the other hand, no significant benefit was obtained from the SA subscale for this Group. Now, 83.33% (15) of Group 2 users presented effective benefits in subscales FC and RA, 72.23% (13) for RV, 5.56% (1) for subscale SA (Figure 2).

In the Global assessment, a real performance improvement, that is, an index with hearing aid that was 10% better than the index without hearing aid in the three subscales FC, RV, and RA was seen in 75% (6) of those patients from Group 1; 2 users had benefits above 10%, in 82.35% (14) of the individuals who made up Group 2, and the other two presented lower benefits only in subscale RV, and a third one without significant benefits in the three subscales (Figure 3).

Thus, the APHAB questionnaire proved to be an excellent tool, not only o assess the benefit obtained from the use of amplification, but also to predict and confirm the user’s performance when in difficulty of communication in different situations, which still remain despite the use of amplification, thus contributing in adjusting amplification throughout the process, because as we can see in this study, the rehabilitation process is directly linked to the expectations and perceptions of the user herself, and this is the keystone in the hearing aid fitting process.

The aim of the hearing aid fitting process is to offer environment sound amplification and mainly the sounds of speech in a satisfactory and proper manner. Nonetheless, even with amplification, some communication difficulties may depend on hearing loss type, level and configuration, as well as disabilities and auditory handicap experienced by the patient. Therefore, such aspects not only have to be taken into account, but also challenged together with the future user, so that a high expectation with rehab does not impair the real benefit obtained with amplification.

Once again, we stress that the use of these protocols in the pre and post fitting periods, since they proved to be excellent predictors of the difficulties faced by the users of hearing aids, could also help in adjusting the device based on the user’s own perception, however in a quantified and standardized way, often times by means of spontaneous questions - these users feel intimidated or unable to reveal their own difficulties.

By the same token, even if the prescribed acoustic gain values are reached or the speech recognition tests show improvements in speech recognition, the self-assessment questionnaires must be used, since an increase in hearing and/or speech recognition do not guarantee a reduction in the hearing disability handicap the patient experienced. We also consider that the education and advice stage, as well as long term advising, is mandatory to assure a successful rehabilitation, soothing out the difficulties that may eventually slow down treatment progress.

## CONCLUSION

At the end of this study, a critical appreciation of the results led us to conclude that:

1. The self-assessment questionnaires proved to be excellent predictors of the difficulties faced by the users of hearing aids, and they could also help in hearing aid fitting;
2. Significant differences between the groups studied in the self-assessment HHIE S and HHIA protocols, as well as in the APHAB were not found, except in the ease of communication subscale, in which Group 1, without complaints related to amplification characteristics obtained greater benefits.
